# Multidimensional evaluation of soil pollution from railway tracks

**DOI:** 10.1007/s10646-015-1426-8

**Published:** 2015-03-12

**Authors:** Małgorzata Wierzbicka, Olga Bemowska-Kałabun, Barbara Gworek

**Affiliations:** 1 Faculty of Biology, University of Warsaw, I. Miecznikowa 1, 02-096 Warsaw, Poland; 2 Institute of Environmental Protection – National Research Institute, Krucza 5/11d, 00-548 Warsaw, Poland

**Keywords:** Railway, Railway tracks, Soil, Pollution, Biotests

## Abstract

Railway transport is a source of pollution to soils and living organisms by e.g. PAHs, PCBs, oil-derived products, pesticides and heavy metals. Soil toxicity evaluation requires chemical analyses, indicating the type and content of particular pollutants, as well as biological analyses, which allow assessing the reaction of organisms to these pollutants. This paper is focused on a multi-aspect evaluation of the degree of toxicity and pollution of soil in selected railway areas from north-eastern Poland by application of numerous biotests and chemical analyses. The soils were sampled on railway tracks from the following railway stations: Białystok Fabryczny, Siemianówka, Hajnówka, Iława Główna and Waliły. The most toxic soils occur on the railway tracks at Białystok Fabryczny and Siemianówka. They had a significant toxic effect on test organisms from various trophic levels. The contents of PAHs, PCBs, heavy metals, oil-derived hydrocarbons and pesticide residues were determined in the examined soils. In all cases the detected pollutants did not exceed the admissible levels. The highest content of oil-derived substances was noted in soils from Białystok Fabryczny and concentrations were moderate in soils from Siemianówka. Although the pollutants determined in soils from railway tracks did not exceed the admissible values, they had a toxic effect on numerous test organisms from different trophic levels. This suggests a synergistic effect of low concentrations (within the admissible levels) of several pollutants together, which resulted in a toxic effect on the organisms. Thus, there is a strong need of not only chemical, but also ecotoxicological analyses during the evaluation of environmental conditions. Based on data obtained from biological and chemical analyses, we concluded that railway transport may pose a hazard to the natural environment to a larger extent that hitherto expected.

## Introduction

Soil is one of the most important elements of the natural environment. It is not only the main link in the cycling of elements but also the basic component of the trophic system: soil–plant–animal–human being (Kabata-Pendias and Pendias 1999). Railway transport may be a potential source of pollution in soils. Hazard caused by the functioning of the railway infrastructure is practically connected with all environmental components—lithosphere, hydrosphere, atmosphere and biosphere. Toxic substances polluting the soil in the vicinity of railways may be transported from soils to plants occurring near the railway tracks and to groups of living organisms. The variable use of railway areas results in the large variability of substances polluting soil and plants along railway tracks. This group of pollutants includes PAHs, PCBs, herbicides, fungicides, insecticides, oil-derived substances (e.g. mineral oils) and heavy metals (Binkiewicz 2005; Burkhardt et al. 2008; Galera et al. 2011; Liu et al. 2009; Malawska and Wiłkomirski 1999, 2000, 2001a, 2001b; Moret et al. 2007; Schweinsberg et al. 1999; Thierfelder and Sandström 2008; Wiłkomirski et al. 2011, 2012; Zhang et al. 2012).

Most studies on environmental pollution by railway transport are focused on the type and concentration of the polluting agents. In the case of living organisms, populations or biocoenoses it is difficult to predict their reaction to pollutant toxicity based only on physical and chemical parameters. Toxic compounds may occur in many chemical forms with different bioavailability. Organismal reactions may result from the effect of particular compounds as well as the synergistic effect of all substances polluting a given area (Persoone et al. 2003; Traczewska 2011; Walker et al. 2002; Zimny 2006).

Therefore, environmental monitoring focused on collecting data on the pollution of environmental components and predicting its effects commonly uses bioindicative studies as one of the methods of environmental assessment (Traczewska 2011; Zimny 2006). Bioindicative methods include e.g. biotests, which are biological tests that determine the presence of toxic substances in the environment as well as evaluate their toxicity by measuring the influence of various substances on living organisms. Biotests allow determining the degree of alteration of the environment and its particular components. Along with chemical measurements they represent an indispensable base to obtain a full image of the studied problem (Hund-Rinke et al. 2002; Keddy et al. 1995; Kuczyńska et al. 2003, 2005; Persoone et al. 2003; Põllumaa et al. 2004; Rojíčková-Padrtová et al. 1998; Traczewska 2011; Wolska et al. 2007).

This paper is focused on the multi-aspect evaluation of the degree of toxicity in soils from railway areas using a battery of biotests (plant test Phytotoxkit, animal tests Ostracodtoxkit and Daphtoxkit, bacterial test Microtox) combined with chemical analyses. The examined soils were collected from railway tracks located in north-eastern Poland at the Białystok Fabryczny, Waliły, Hajnówka, and Siemianówka stations, representing the Podlasie area, and the large station at Iława Główna, representing the western part of the Masuria region. This paper is a continuation of earlier studies focused on the pollution of railway areas in north-eastern Poland (Galera et al. 2011, 2012; Wierzbicka et al. 2014; Wiłkomirski et al. 2011, 2012).

## Materials and methods

### Soils from railway tracks

**Fig. 1:**
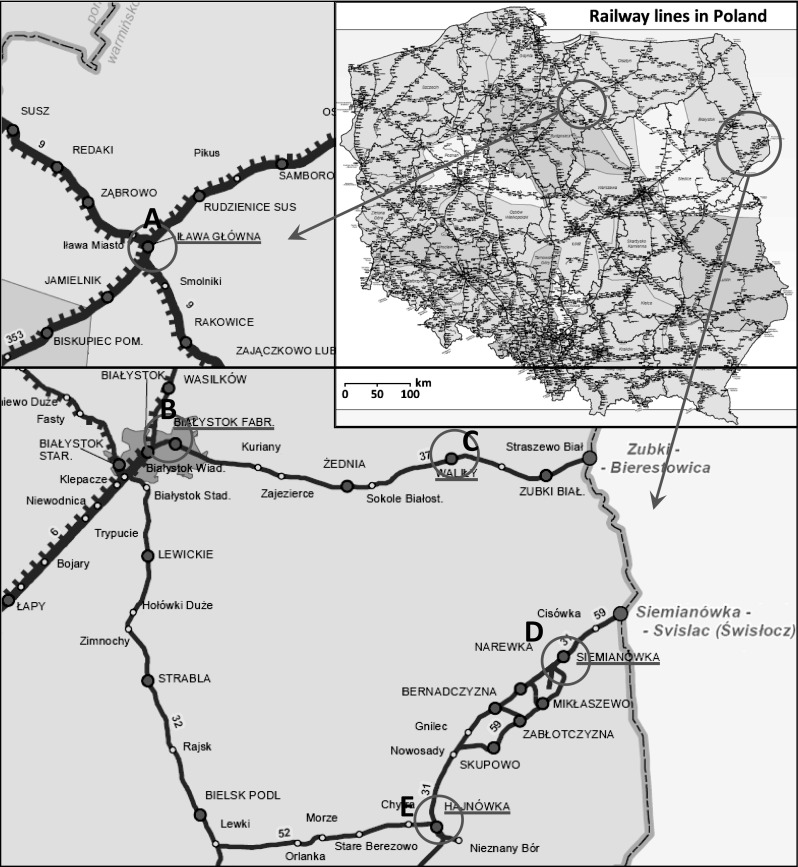
Location of soil sampling sites on railway tracks. The stations are located in north-eastern Poland: Iława Główna **a**, Białystok Fabryczny **b**, Waliły **c**, Siemianówka **d** and Hajnówka **e**

The examined substrates, which were referred to as “railway basement soils” by Wiłkomirski et al. (2011), were collected from the depth of 0–20 cm under the crushed stone (“breakstone” in Wiłkomirski et al. 2011), occur near railway tracks at numerous railway stations in north-eastern Poland (Fig. 1). The stations Białystok Fabryczny and Waliły (line no. 37), as well as Hajnówka and Siemianówka (line no. 31) are located in the Podlasie region. Railway lines nos. 37 and 31 passing through these stations are connected with line no. 32 and form a closed system (Fig. 1). The railway subsoils were also sampled within the large station at Iława Górna, which is located in the western part of the Masuria region (Fig. 1). The station is an important junction of railway lines (lines nos. 9, 251 and 353). The soils were collected in 2007–2008, at that time all stations were active. General data on the stations where the soils were sampled are presented in Table 1.

**Table 1:** General data on the railway stations where the soils were sampled for analysis

Station	Status	Surroundings	Coordinates
Białystok Fabryczny, track 34	Active; cargo transport; low transport intensity	Track located on a 2-m high embankment; poor substrate located on gravel and wooden ties; rare vegetation, composed of grasses and *Geranium robertianum*	53°08′25″N 23°11′02″E
Siemianówka	Active; cargo transport	One track; station area surrounded by ruderal species	52°54′03″N 23°49′07″E
Hajnówka, switch	Active; cargo transport	Poor vegetation cover on the active track; rich vegetation on the closed-down tracks; ruderal species around the station	52°44′02″N 23°35′00″E
Iława Główna (2 samples: siding and bottle-washer)	Active; cargo and passenger transport	Junction covers area of ~2 km^2^; the surroundings encompass: platforms, buildings of the passenger station, railway siding, transfer site, bottle-washer; important junction of railway tracks connecting the west with east and the north with the south of Poland	53°34′58″N 19°34′27″E
Waliły	Currently closed-down; earlier cargo transport	Track substrate composed of broken stone and concrete ties; abundant *G. robertianum* in the station surroundings	53°06′29″N 23°38′51″E

Material analysed in this study was a composite of several soil samples (15–20) collected between the rails and beyond the rails in a single railway track on a given station (or a given station area), according to the procedure described by Wiłkomirski et al. (2011). In the case of Białystok Fabryczny, Waliły, Hajnówka, and Siemianówka, the collected soils were treated as single samples from a given station. In the case of Iława Górna, the soils were collected near the rails at the railway siding and bottle-washer, and were treated as two separate samples from this station. The basic soil properties of the railway subsoils from this region were described in Table 6 after Galera et al. (2011), which was a part of our previous research.

### Ecotoxicological analyses

The toxicity of the examined railway subsoils was evaluated using the following biotests: Phytotoxkit™, Ostracodtoxkit F™, Daphtoxkit F™ and Microtox (Table 2).

**Table 2:** General characteristics of the applied biotests

Test	Phytotoxkit™	Ostracodtoxkit F™	Daphtoxkit F™	Microtox
Test organism	Plants: *Lepidium sativum*, *Sinapis alba*, *Sorghum saccharatum*	Crustaceans: *Heterocypris incongruens*	Crustaceans: *Daphnia magna*	Bacteria: *Vibrio fischeri* (*Photobacterium phosphoreum*)
Final points of toxicity measurements	Root growth inhibition	Mortality; growth inhibition	Mortality	Bioluminescence inhibition
Exposure time	7 days	6 days	72 h	5 and 15 min
Test type	Chronic	Chronic	Acute	Acute
Standards	Analogous to ISO^a^ 11269-1	Approval by ISO pending	In accordance with: OECD Guideline 202 and ISO 6341	In accordance with: ISO, DIN^c^, EPA^d^, AFNOR^e^, ASTM^f^
Repetitions	5	6	4	2
Control batch	Reference soil, in accordance with OECD^b^ (for soil toxicity tests)	Standardized sand which is part of the experimental kit	Hydrobiological medium (water from *Daphnia* breed with algae addition)	2 % NaCl solution

^a^ISO (*International Organization for Standardization*)

^b^OECD (*Organization for Economic Cooperation and Development*)

^c^DIN (*Deutsches Institut für Normung*)

^d^USEPA/EPA (*United States Environmental Protection Agency*)

^e^AFNOR (*Association Française de Normalisation*)

^f^ASTM (*American Society for Testing and Materials*)

Phytotoxkit is a plant test using *Lepidium sativum* L., *Sinapis alba* L. and *Sorghum saccharatum* (L. emend. L.) Moench as the test species. The effectiveness of these species as bioindicators is confirmed in literature (Traczewska 2011; Czerniawska-Kusza et al. 2006). Phytotoxkit is a short-term chronic test, measuring the inhibition of root growth (Table 2). 90 cm^3^ of each examined soil or reference soil were added to the test plates; the reference soil was a standardized control soil supplied by the test producer (similar to OECD standardized artificial soil for tests with invertebrates; comprising sand, kaolin, and peat; the reaction was regulated with calcium carbonate). Particular soils were inserted on test plates and soaked with distilled water according to the water capacity determined for each examined soil. Soils on the plates were covered with filter paper. Next, ten seeds of each test plant were arranged in one line and at equal distances from each other on each plate. The test plates were closed, vertically arranged in stands and inserted in an incubator. The seeds were incubated in darkness at 25 °C for 7 days. The test plates were imaged every 24–72 h using a computer scanner. Root length measurements were made using Image Tool software. The obtained results allowed determining the root toxicity index [(A − B)/A]×100, where: A—root length on the reference soil; B—root length on the examined soil (Phytotoxkit. Standard operational procedure). The test was repeated five times for each of the three mentioned species (five test plates for 10 seeds—a total of 50 seeds of each species for each of the examined soils). The method evaluating root growth inhibition is useful for toxicity analysis of chemical compounds, excluding volatile substances or those influencing photosynthesis (ISO 11269-1:^1993^; PN-ISO 11269-1:^1998^; Traczewska 2011).

Selected plant species included both monocots (*Sorghum saccharatum*) and dicotyledonous plants (*Lepidium sativum* and *Sinapis alba*), which are recommended by the manufacturer of the biotest. These plant species are often used in assays of phytotoxicity due to their very rapid germination, growth of roots and shoots, which enables the observation and reading the results after 3 days of the test. In addition, germination of seeds was checked before the test. For *Lepidium sativum*, 100 % germination was achieved, whereas *Sinapis alba* and *Sorghum saccharatum*, 97 and 92 %, respectively. Therefore, all seeds were found to be suitable for performance of the Phytotoxkit biotests. In order to perform the Phytotoxkit biotest correctly, there is a need to follow the manufacturer’s instructions. The Test should be performed using at least three different plant species. The incubation period of test plants with the contaminated soil should last at least 3 days (in our study, observations were conducted for 7 days). Incubation should be carried out in the dark, at 25 °C. The test should be performed with at least three repeats of each experiment variant (including controls). In our study the test was repeated five times for each used species. The results for tested samples refer to the results obtained in the control group. These assumptions have been realized in the present study. Phytotoxkit kit contains standard materials and biomaterials, which provides a very high reproducibility of the results (Phytotoxkit. Standard operational procedure). The manufacturer does not specify additional validity criteria of the test (other than following the manual).

Ostracodtoxkit and Daphtoxkit tests serve to evaluate the water and soil condition after transforming the samples into soil extracts (Hund-Rinke et al. 2002; Oleszczuk 2008; Płaza et al. 2005, 2010; Põllumaa et al. 2004).

Ostracodtoxkit F™ is a chronic animal test, using *Heterocypris incongruens* as the test organism. The final points of toxicity measurements include mortality and growth inhibition (Table 2). The control batch was the Ostracodtoxkit control sediment—standardized sand supplied by the test producer. Cysts of *H. incongruens* were incubated at 25 °C, in continuous lighting (3000–4000 lx). After incubation, the young organisms were fed with a Spirulina medium supplied by the test producer. The length of young organisms was determined directly after hatching. The examined soils, algal medium (*Scenedesmus* sp.) and test organisms were inserted in multi-pit test plates—each plate for each examined soil. Ten individuals of *H. incongruens* were transferred to each of the six pits of each plate, which represented a separate repetition. The plates were covered with parafilm, sealed with covers, inserted in an incubator and incubated at 25 °C in darkness for 6 days. After this period, mortality was determined and the length of the test organisms was measured once again. Based on the obtained data, the average mortality of the test organisms for every sample and the medium growth inhibition in percentage of the control batch were calculated (Ostracodtoxkit F. Standard operational procedure). The test was repeated six times for each soil and a total of 60 organisms was used in each variant.

In order to perform the Ostracodtoxkit biotest correctly, there is a need to follow the manufacturer’s instructions. The incubation period of test organisms with contaminated samples should last 6 days. Incubation should be carried out in the dark, at 25 °C. At least six repeats of each experiment variant should be performed (including controls). The results for tested samples refers to the results obtained in the control group. These assumptions have been realized in the present study. Ostracodtoxkit kit contains standard materials and biomaterials, which provides a very high reproducibility of the results. To perform the Ostracodtox biotest properly, the average growth in length of the control organisms must be at least 400 µm. In addition, the average percentage of mortality of the test organisms in the control should not exceed 20 % (Ostracodtoxkit F. Standard operational procedure). For this study the mean growth in length of control organisms was 710.47 µm and the average percentage of mortality of control organisms was slightly above 20 %.

Daphtoxkit F™ is an animal test measuring acute toxicity and using *Daphnia magna* as the test organism. The final point of the toxicity measurement is organism mortality (Table 2). The test allows determining the LC_50_ or EC_50_ values. The biotest was carried out in multi-pit test plates. Water extracts of the examined soils were used in the test. The control batch represented a special hydrobiological medium (water after the growth of test organisms with addition of algae). The first test was carried out with 100 % water soil extracts (10 g of the examined soil per 50 ml distilled water) and next, based on the obtained results, the test was conducted with dilutions of the soil extracts (100; 50; 25; 12.5 and 6.25 %) for the most toxic media. In both tests, 10 ml of extracts prepared from the examined soils were transferred to the test pits. In the case of tests with dilutions, subsequent dilutions were prepared by addition of 100 % soil extract of the examined soil and distilled water in relevant proportions. Similarly were prepared dilutions of the control batch. Five young individuals of *D. magna* of comparable sizes were inserted in each test pit (four test pits per each soil). Prior to the tests the organisms were fed with an algal suspension. The test plates were covered with parafilm, sealed and incubated in darkness at 20 °C for 3 days. After 24, 48 and 72 h of incubation, the dead or immobilized organisms were counted. Organism mortality and toxicity index were calculated [(A−B)/A]×100, where: A—number of live organisms in the control batch; B—number of live organisms in the examined samples (Daphtoxkit F ™ *Magna*. Standard operational procedure). The tests were repeated four times and a total of 20 organisms was used for each examined soil extract with a given dilution.

To perform the Daphtoxkit biotest correctly, there is a need to follow the manufacturer’s instructions. The incubation period of test organisms with analyzed samples should last 48 h (in this test, time of exposure was extended to 72 h in order to observe further changes). Incubation should be carried out in the dark, at 20–25 °C. At least three repeats of each experiment variant should be performed (including controls). The results for tested samples refer to the results obtained in the control group. These assumptions have been realized in the present study. Daphtoxkit kit contains standard materials and biomaterials, which provides a very high reproducibility of the results. To perform the Daphtoxkit biotest properly, the number of killed and immobilized organisms should not exceed 10 % in the control (Daphtoxkit F™ Magna. Standard operational procedure). For this study the average mortality of control organisms after 24 and 48 h was 0 %, whereas after 72 h the mortality reached 30 % (during the test, organisms were not fed, therefore the extension of the test to 72 h disrupted somewhat obtained result for the third day due to starvation).

Microtox is a very sensitive test of acute toxicity, using the marine bacteria *Vibrio fischeri* as the test organism. The final point of the test is the inhibition of bacterial bioluminescence (Table 2). For testing solid samples are applied: the Microtox Basic Solid Test (screening test) and the Microtox Solid Phase Test (dilution test), which use soil extracts. The control batch was a 2 % NaCl solution (recommended by the producer). The first test was conducted with 100 % soil extracts (7 g of the examined soil per 35 ml of a 2 % NaCl solution); next, based on the obtained results a test with dilutions of the examined soils was carried out (100; 50; 25; 12.5; 6.25; 3.125; 1.56; 0.78; 0.39; 0.195; 0.098; 0.049 and 0.02 %) for the most toxic media. Subsequent dilutions of the examined extracts were obtained using a 2 % NaCl solution. Prior to the tests, a bacterial suspension was prepared by adding a restored solution to the lyophilized bacteria. Samples with the examined solutions were inserted in an incubator cooled to about 15 °C and integrated with a photometer (Microtox M500 analyser). 200 μl of a suspension of the restored bacteria were added to each sample. Photometer calibration was conducted in accordance with the test procedures. The results were noted after 5 and 15 min after starting the test. The obtained results were presented in form of a percentage effect of illumination inhibition after 5 and 15 min in relation to the control batch. EC_50_ values were also determined (Ghirardini et al. 2009; Microtox^®^ M500. Industry-leading toxicity detection). The test was repeated twice.

To perform the Microtox biotest correctly, there is a need to follow the manufacturer’s instructions. Reading of results should be made after 5 and 15 min of incubation test organisms with contaminated samples. Incubation should be carried out at 15 °C. The measurement should be performed in duplicate. According to the manufacturer, the pH of samples should be in the range of 6.5–8.5. Within these limits, the bacteria *Vibrio Fischeri* show optimal light production (Microtox^®^ M500. Industry-leading toxicity detection). These assumptions have been realized in the present study. Microtox kit contains standard materials and biomaterials, which provides a very high reproducibility of the results.

Statistical analysis for results from biotests was conducted using STATISTICA software. The non-parametric Kruskal–Wallis test was used (for many independent samples). The significance level was at α = 0.05.

All validity criteria for each biotest can be found in the cited manuals. Results of all tests complied with validity criteria specified by test protocols.

### Chemical analyses

Based on the biotest results, three soils were selected for further chemical analysis (two most toxic from Białystok Fabryczny and Siemianówka and additionally a soil from Waliły). The studies were conducted in accredited laboratories (Accreditation Certificates of the Scientific Laboratory no. AB 463 and AB 757, complying with requirements of the norm PN-EN ISO/IEC 17025:^2005^ in accordance with EN ISO/IEC 17025:^2005^). The soil samples were tested for the presence of the following compounds:

- PAHs: naphthalene, phenanthrene, anthracene, fluoranthene, chrysene, benz[a]anthracene, benz[a]pyrene, benz[a]fluoranthene, benz[ghi]perylene;
- PCBs: congeners 28, 52, 101, 118, 138, 153, 180;
- heavy metals: Cu, Pb, Cd, Zn, Cr, Ni and Hg;
- oil-derived hydrocarbons: sum of petrols (C_6_–C_12_) and mineral oils (C_12_–C_35_);
- residues of pesticides [188 products from the herbicide group (e.g. alachlor, cyanazine, nitrofen), insecticides (e.g. aldrin, heptachlor, carbaryl), fungicides (e.g. bitertanol, metalaxile, pyrazophos) and acaricides (e.g. bromopropylate)].

PAHs and oil-derived hydrocarbons were detected using gas chromatography with a flame ionization detector (GC-FID). PCBs were analysed by application of gas chromatography with electron capture detector (GC-ECD). Heavy metals were analysed using flame atomic absorption spectroscopy (FAAS). The samples were tested also for the presence of pesticide residues with application of gas chromatography with mass spectrometer (GC–MS).

During analysis of the content of PAHs, PCBs, oil-derived hydrocarbons and heavy metals, the soil samples were prepared according to the methodology used in the laboratory, based on international and national methods, such as e.g.:

- PAHs—PN-ISO PN-ISO 18287:^2008^ (in accordance with ISO 18287:^2006^);
- PCBs—DIN 38407-2:^1993^ and US EPA 8082;
- heavy metals—PN-ISO 11466:^2002^ (in accordance with ISO 11466:^1995^) and PN-ISO 11047:^2001^ (in accordance with ISO 11047:^1998^);
- sum of hydrocarbons—PN-EN ISO 9377-2:2002 (in accordance with EN ISO 9377-2:2000), EN 14039:^2005^, EN ISO 6974-1:^2012^.

Analysis of the sum of PAHs and oil-derived hydrocarbons was conducted with use of a gas chromatographer SHIMADZU 2014 with a flame ionization detector (FID). Heavy metals were determined using the atomic absorption spectrometer Solaar. PCBs were tested with application of a gas chromatographer with an electron capture detector (ECD). The calculations were conducted using standard procedures of quantitative analysis—i.e. calibration curve and peak area calculations.

In the pesticide analysis, the soil samples were extracted in accordance with the QuEChERS method, which is the standard method in such analysis. The method includes the extraction of a 10 g sample with 10 ml acetonitrile. The extraction was conducted in the presence of a citrate buffer. Next, water was removed from the extract using magnesium sulphate and purified in a dispersive phase with application of PSA (adsorbent containing primary and secondary amines). After purifying, the extracts were analysed using Agilent GC 6850Series gas chromatographer with mass detector 5973 MSD (Anastassiades et al. 2003a, 2003b). Pesticide residues were analysed according to the methodology in PN-EN 12393-1,3 + I-01/PN-EN 12393-1,3 (method PN-EN 12393:^2009^, PN-EN 12393-1:^2009^, PN-EN 12393-3:^2009^; in accordance with EN 12393:^2008^, EN 12393-1:^2008^, EN 12393-3:^2008^). The analyses were conducted based on method PN-EN 15662:^2008^ (in accordance with EN 15662:^ 2008^).

## Results

### Phytotoxkit biotest

**Fig. 2:**
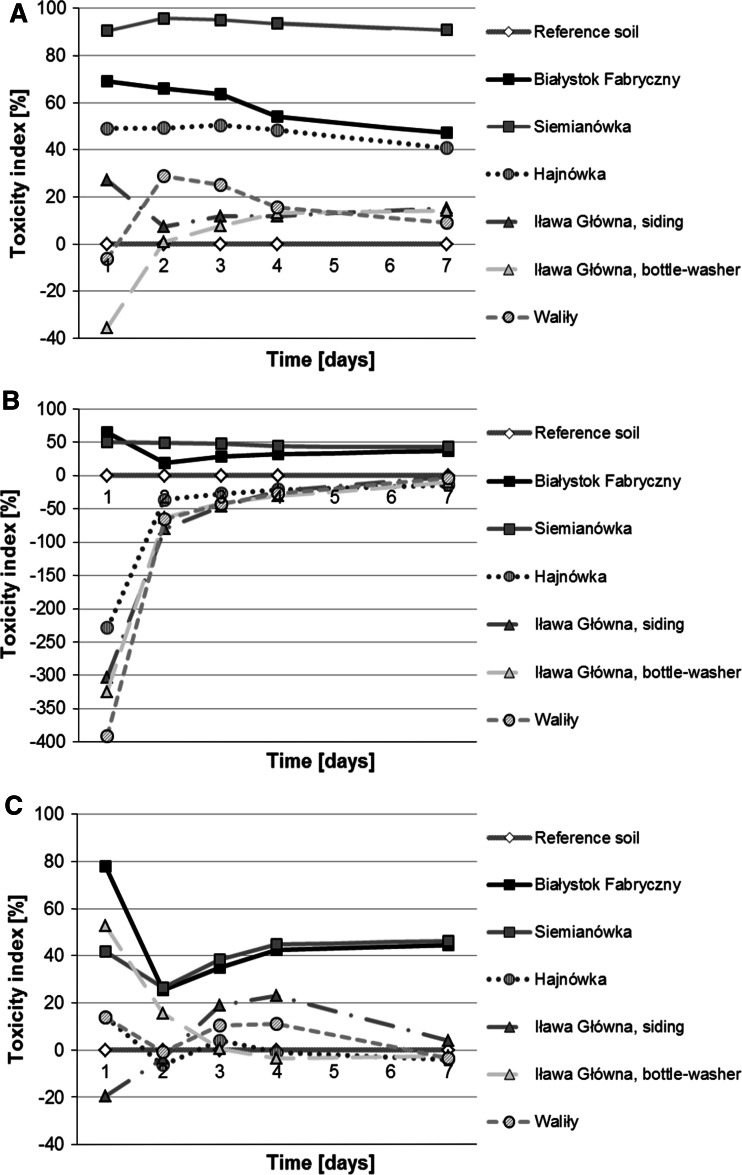
Root toxicity index for *Lepidium sativum* **a**, *Sinapis alba* **b** and *Sorghum saccharatum* **c**. Higher percentage of the toxicity index indicates a higher toxicity of the substrate

**Fig. 3:**
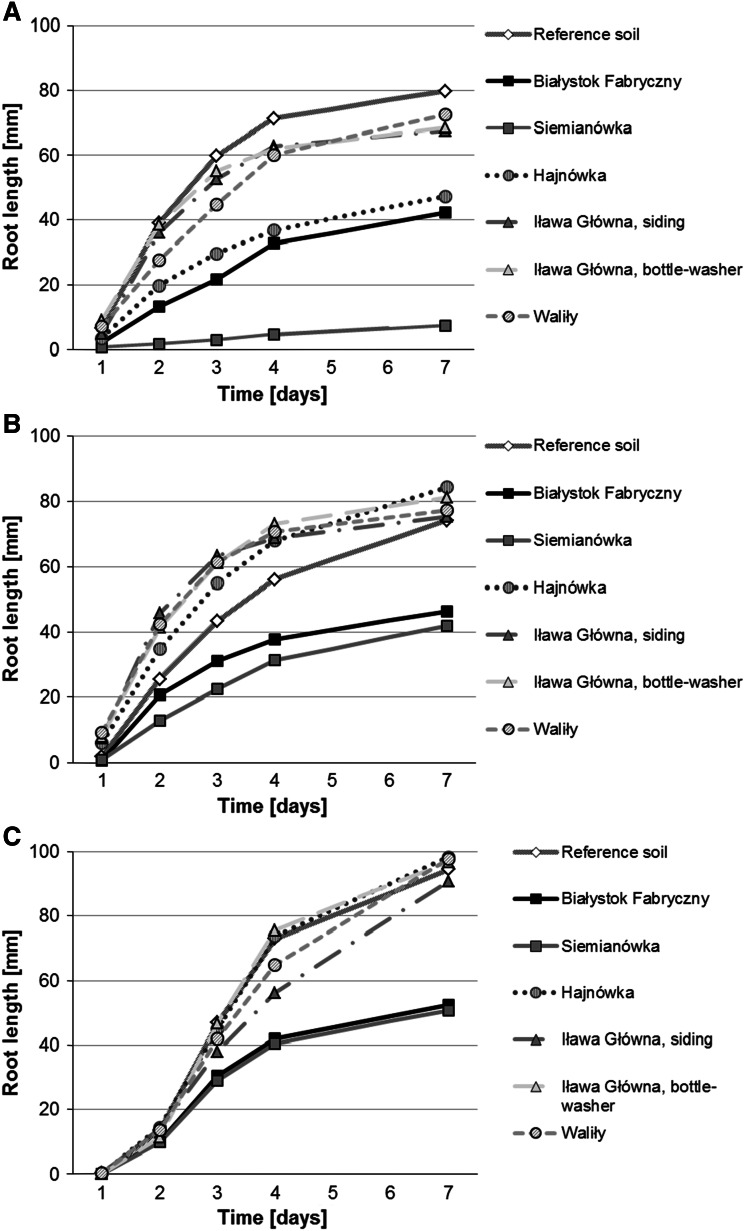
Root length in *Lepidium sativum* **a**, *Sinapis alba* **b** and *Sorghum saccharatum* **c** grown on soils from railway tracks and on the reference soil

In the Phytotoxkit text, the root toxicity index was the highest for soils from railway tracks at Siemianówka (~90 % with *L. sativum*, ~50 % with *S. alba* and 26–46 % with *S. saccharatum*) and Białystok Fabryczny (50–70 % with *L. sativum*, 18–65 % with *S. alba* and 25–78 % with *S. saccharatum*). Soils from Siemianówka and Białystok Fabryczny were the most toxic to the applied test organisms (Fig. 2). Seedlings incubated on these media were characterized by the lowest root growth (Fig. 3) and the obtained differences of root growth were significant in relation to the control batch (Table 3).

**Table 3:** Statistical analysis of the results of root growth for *L. sativum*, *S. alba* and *S. saccharatum* on soils from railway tracks in relation to the control reference soil

Test organism	Station																													
	Białystok Fabryczny					Siemianówka					Hajnówka					Iława Główna, siding					Iława Główna, bottle-washer					Waliły				
	Day					Day					Day					Day					Day					Day				
	1	2	3	4	7	1	2	3	4	7	1	2	3	4	7	1	2	3	4	7	1	2	3	4	7	1	2	3	4	7
*L. sativum*	+	+	+	+	+	+	+	+	+	+	+	+	+	+	+	−	−	−	−	−	−	−	−	−	−	−	+	+	−	−
*S. alba*	−	−	−	+	+	−	+	+	+	+	+	−	−	−	−	+	+	+	−	−	+	+	+	+	−	+	+	+	−	−
*S.* *saccharatum*	−	−	+	+	+	−	−	+	+	+	−	−	−	−	−	−	−	−	+	−	−	−	−	−	−	−	−	−	−	−

The numbers refer to the subsequent days of Phytotoxkit test duration. A plus (+) refers to statistically significant differences and a minus (−) refers to results insignificant in relation to the control soil. Statistical analysis was conducted using STATISTICA software. The non-parametric Kruskal–Wallis test was used (for many independent samples). The significance level was at α = 0.05

Soils from railway tracks at the remaining stations were characterized by much lower root toxicity index in the case of all applied test species (Fig. 2). These soils indicated a low toxicity for the applied species or even its lack. The most sensitive bioindicator was *L. sativum*, next *S. alba*, and the least *S. saccharatum*.

### Ostracodtoxkit biotest

**Fig. 4:**
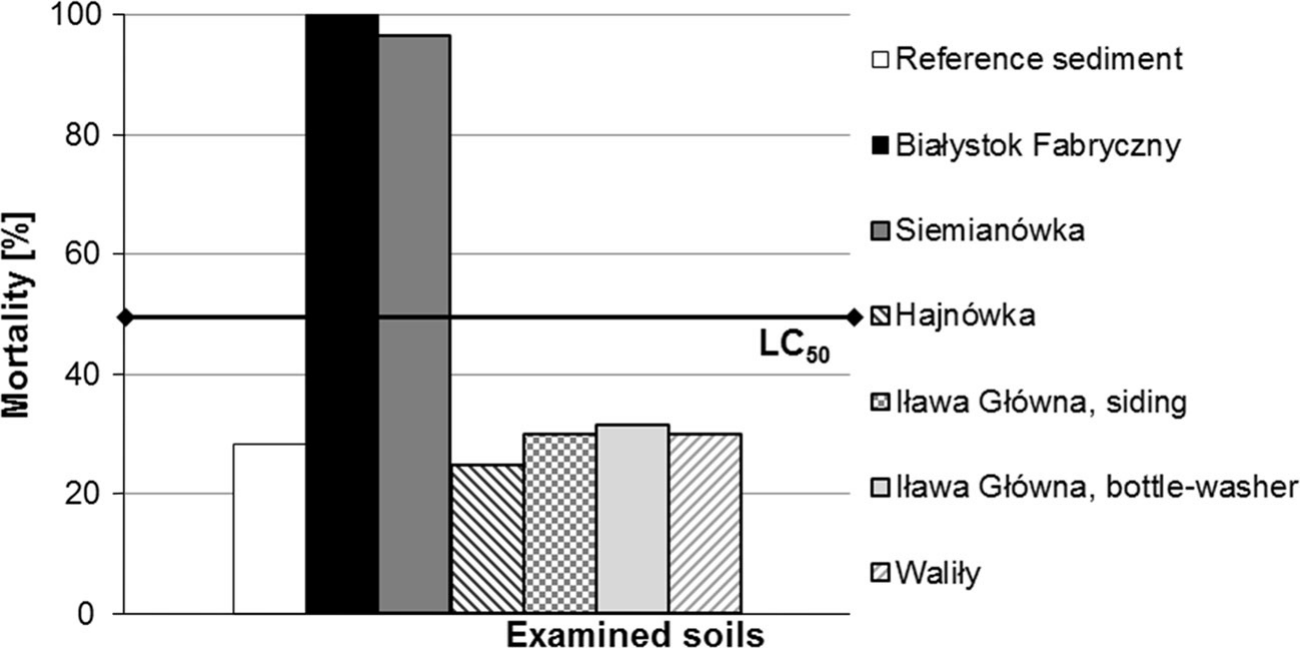
Mortality of test organisms (*Heterocypris incongruens*) incubated on the examined soils; *black line* refers to the LC_50_ effect

In the Ostracodtoxkit test, after 6 days of incubation of the crustaceans *H. incongruens* with the examined media, the highest mortality of the test organisms was noted for soils from the railway tracks at Białystok Fabryczny (100 %) and Siemianówka (~97 %). For the remaining soils, organism mortality was at the control level (lack of toxic effect) and reached about 30 % (Fig. 4). Mobility was not inhibited in the test organisms that survived the incubation on soil samples from Siemianówka, Iława Główna, Waliły and Hajnówka.

**Fig. 5:**
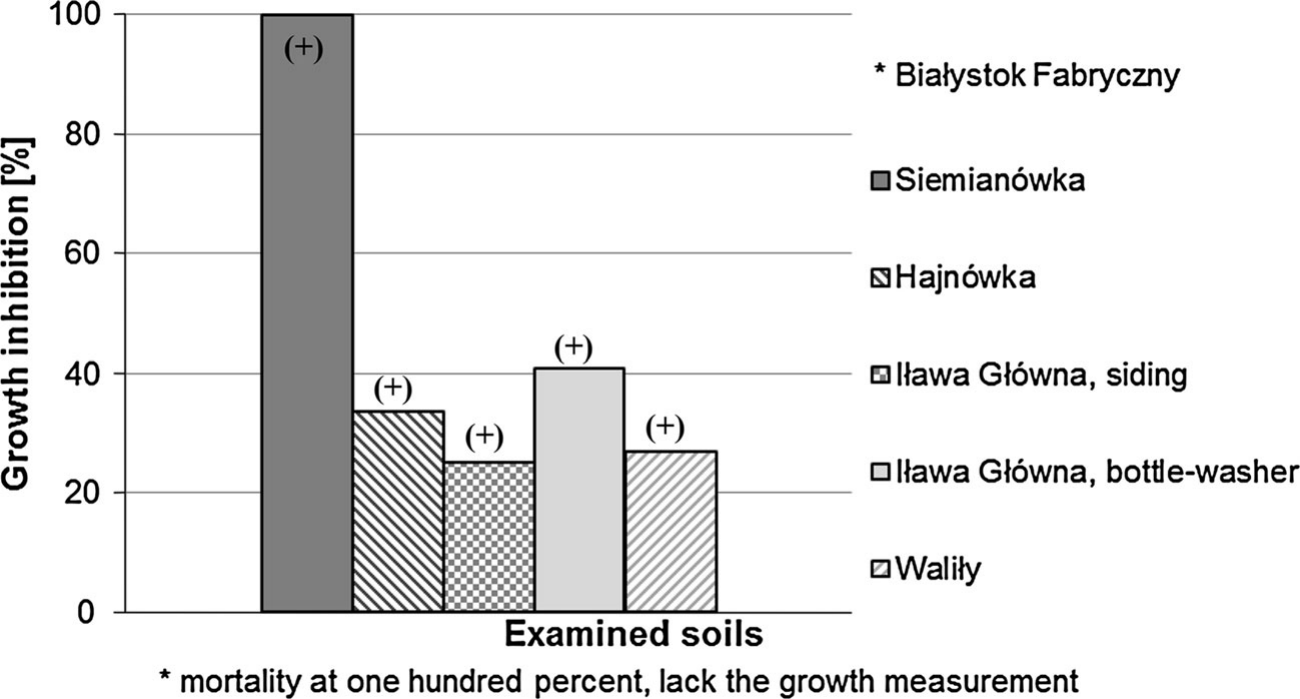
Growth inhibition in test organisms (*Heterocypris incongruens*) incubated on the examined soils in comparison to the reference soil. *Plus* (+) refers to statistically significant differences in comparison to the reference soil. Statistical analysis was conducted using STATISTICA software. The non-parametric Kruskal–Wallis test was used (for many independent samples). The significance level was at α = 0.05

The highest mean percentage growth inhibition took place in the test organisms incubated on soil from the railway tracks at Siemianówka (due to 100 % mortality of the test organisms in the sample from Białystok Fabryczny, growth inhibition could not be measured). Mean percentage growth inhibition in *H. incongruens* in the remaining samples varied within 25 to 40 % in relation to the control batch (Fig. 5). The differences were statistically significant in relation to the control batch, where the mean increase of organism length in the plate was 710.47 μm.

### Daphtoxkit biotest

**Fig. 6:**
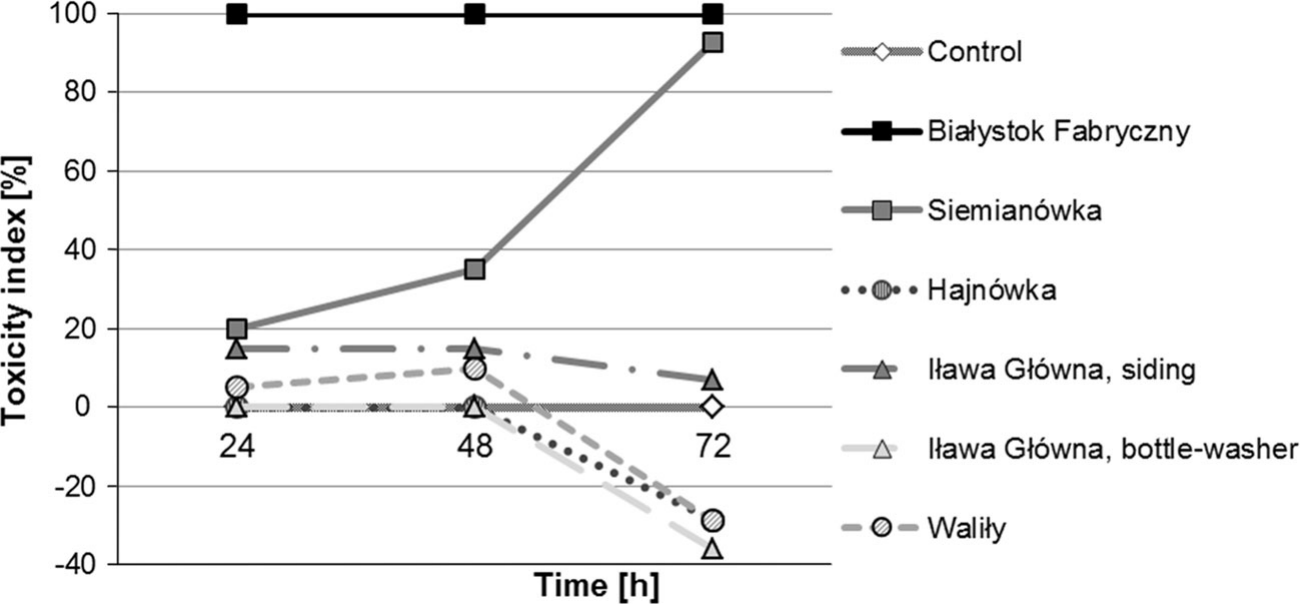
Toxicity index in *Daphnia magna* after 24, 48 and 72 h of incubation in all examined soil extracts; 100 % concentration. Higher percentage of the toxicity index indicates a higher toxicity of the examined substrate

In the Daphtoxkit test, in which 100 % soil extracts were analysed, the toxicity index for *D. magna* in relation to the control batch was the highest for the sample collected from the railway tracks at Białystok Fabryczny (100 % mortality). By the end of the test (after incubation for 72 h), the toxicity index was also high in the sample from Siemianówka, where decreased mobility activity was additionally observed in the incubated organisms. The LC_50_ effect was exceeded for both samples (Fig. 6).

**Fig. 7:**
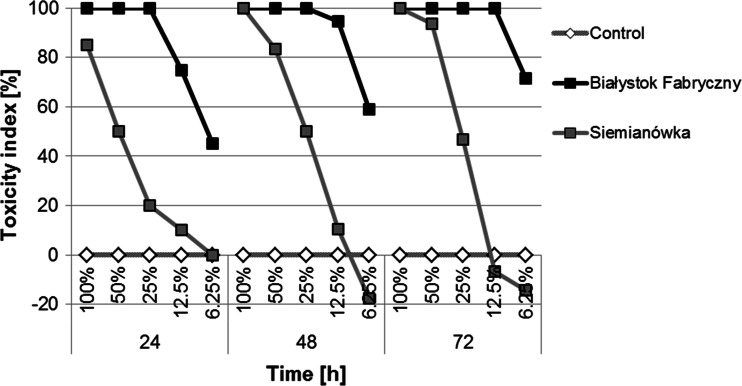
Toxicity index in *Daphnia magna* after 24, 48 and 72 h of incubation in the most toxic extracts—samples from Białystok Fabryczny and Siemianówka (concentrations: 100; 50; 25; 12.5 and 6.25 %). Higher percentage of the toxicity index indicates a higher toxicity of the examined substrate

For further Daphtoxkit tests, a series of soil extract dilutions (100; 50; 25; 12.5 and 6.25 %) were created with soils from railway tracks at Białystok Fabryczny and Siemianówka (Fig. 7). Mortality was 100 % in undiluted (100 %) soil extract from a Białystok Fabryczny sample and soil extracts of 50, 25 and 12.5 % from this sample after 72 h, as well as in undiluted soil extract from a Siemianówka sample after 48 h. The toxicity index was higher with a higher concentration of the applied solutions. All concentrations of soil extracts from Białystok Fabryczny and the 100, 50 and 25 % concentrations of the sample from Siemianówka exceeded the LC_50_. The test organisms that survived incubation indicated a decreased activity in the least diluted soil extract from the sample from Siemianówka and a significant decrease of activity in the most diluted soil extracts from the samples from Białystok Fabryczny.

### Microtox biotest

**Fig. 8:**
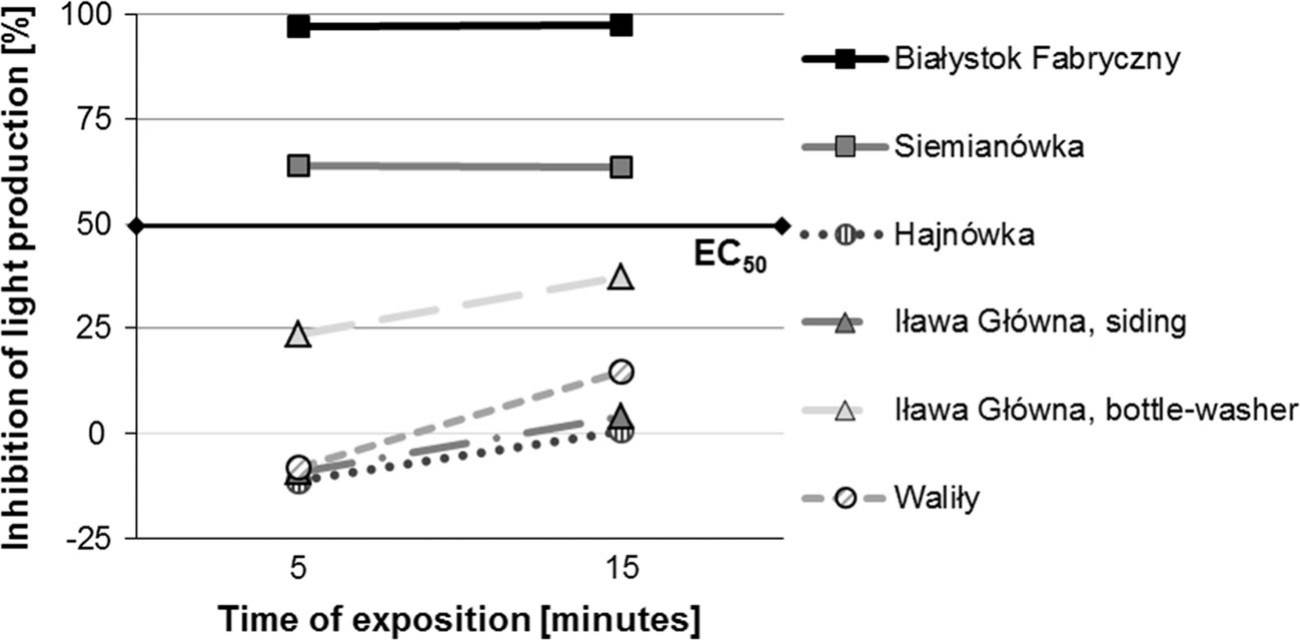
Inhibition of light production in the bacteria *Vibrio fischeri* after 5 and 15 min of exposure to contact with the soil extract in relation to the control batch—all samples, 100 % concentrations. *Black line* refers to the EC_50_ effect

In the Microtox test the largest inhibition of lighting intensity in the bacteria *V. fischeri* (above the EC_50_ value) with undiluted soil extracts (determined as 100 % concentrations) from all samples (Fig. 8) was observed for samples from Białystok Fabryczny (~100 %) and Siemianówka (63 %). The remaining samples did not exceed EC_50_ values; they, however, indicated a more intense inhibition of bacterial luminescence after 15 min than after 5 min. The sample from the bottle-washer at Iława Główna indicated an over 25 % effect of lighting inhibition (medium toxicity). The remaining samples were characterized by the lack of a toxic effect or only an insignificant effect of luminescence inhibition (Fig. 8).

**Fig. 9:**
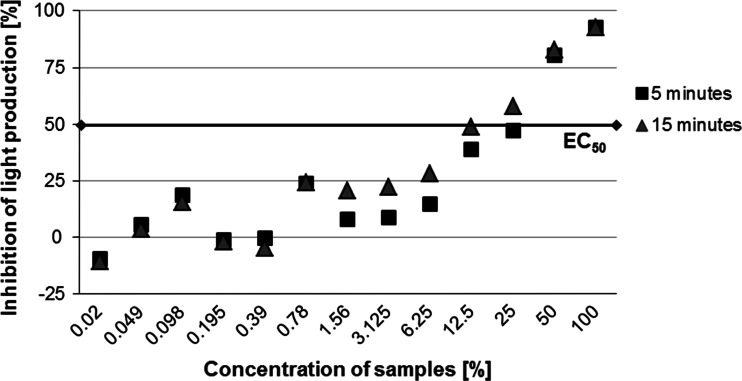
Inhibition of light production in the bacteria *Vibrio fischeri* after 5 and 15 min of exposure to contact with the soil extract in relation to the control batch—sample from the railway tracks at Białystok Fabryczny, at increasing concentrations (from 0.02 to 100 %). *Black line* refers to the EC_50_ effect

**Fig. 10:**
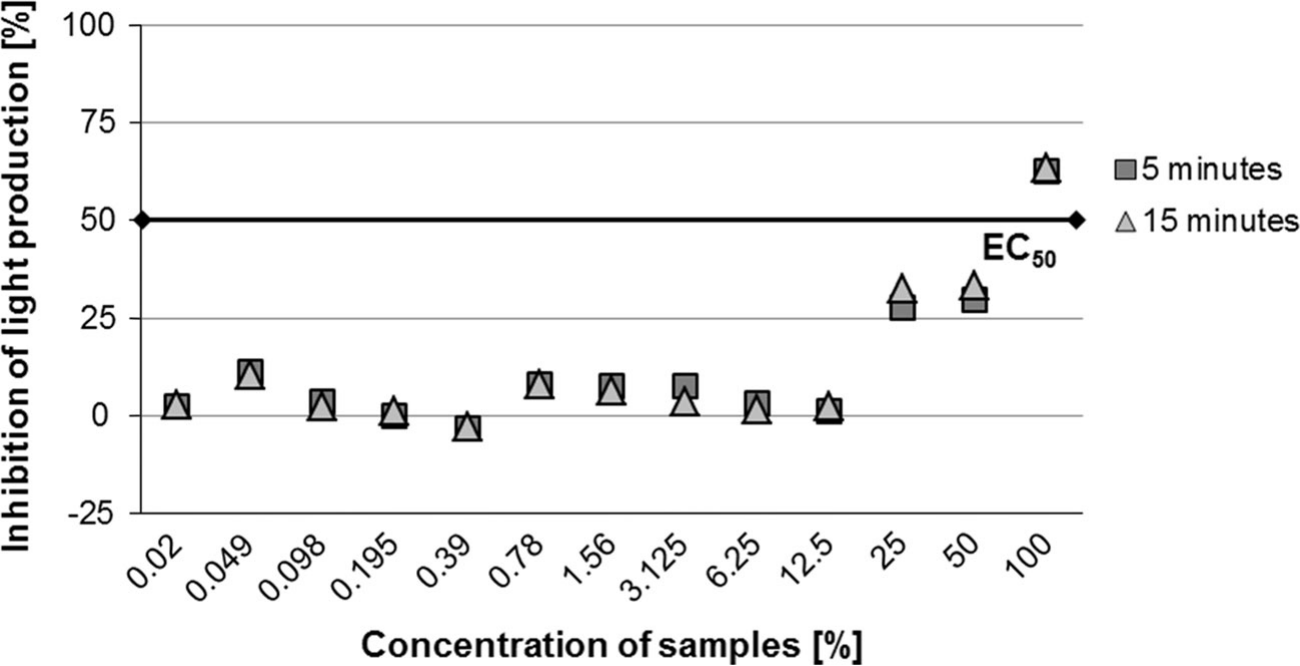
Inhibition of light production in the bacteria *Vibrio fischeri* after 5 and 15 min of exposure to contact with the soil extract in relation to the control batch—sample from the railway tracks at Siemianówka, at increasing concentrations (from 0.02 to 100 %). *Black line* refers to the EC_50_ effect

Samples from Białystok Fabryczny and Siemianówka were selected for further Microtox tests with a series of soil extract dilutions (0.02–100 %). In these tests the highest inhibition of bacteria lighting intensity was observed for the highest concentrations of soil extracts from the sample from Białystok Fabryczny. The EC_50_ value in extracts from this sample was exceeded after 5 min of exposure for 100 and 50 % concentrations and after 15 min for the 25 % concentration (Fig. 9). In the case of soil extracts from the sample from Siemianówka the EC_50_ value was reached after 5 min only for the 100 % concentration. In all remaining extracts (dilutions 0.02–50 %), the EC_50_ value was not exceeded (Fig. 10).

### Chemical analyses

Based on the biotest results, soils from railway tracks at Białystok Fabryczny and Siemianówka and soil from the Waliły station were selected for further chemical analyses. The soils were tested for the presence of PAHs and PCBs (GC-FID method), heavy metals Cu, Pb, Cd, Zn, Cr, Ni and Hg (FAAS method), oil-derived hydrocarbons—sum of petrols and mineral oils (GC-FID method)—and pesticides (GC–MS method) (Table 4).

**Table 4:** Results of chemical analysis of the soil samples collected from the railway tracks at Białystok Fabryczny, Siemianówka and Waliły, as well as the Polish legal laws for soils

Parameter	Station			Polish legal limits for soils^a^
	Białystok Fabryczny	Siemianówka	Waliły	Communication and industrial areas, mining areas
				Depth [mbgs^b^]
				0–2
∑ petrols (C_6_–C_12_)	134.1 ± 40.2^c^	92.1 ± 27.6	13.4 ± 4.0	500^d^
∑ mineral oils (C_12_–C_35_)	2520.0 ± 730.8	1099.0 ± 318.7	831.5 ± 241.1	3000^e^
∑ PAHs	20.5 ± 4.9	3.3 ± 0.8	2.3 ± 0.6	250^f^
∑ PCBs	0.116 ± 0.046	<0.021	<0.021	2^g^
Zinc	130 ± 10.4	75 ± 6.0	106 ± 8.58	1000
Copper	107 ± 16.1	27 ± 4.1	46 ± 6.9	600
Lead	153 ± 27.5	20 ± 3.6	27 ± 4.9	600
Nickel	14 ± 3.4	17 ± 4.1	52 ± 12.5	300
Mercury	0.06 ± 0.01	<0.05	<0.05	30
Cadmium	<0.70	<0.70	<0.70	15
Chromium	25 ± 5.3	15 ± 3.2	70 ± 14.7	500

Sums: petrols (C_6_–C_12_), mineral oils (C_12_–C_35_), PAHs (naphtalene, phenanthrene, anthracene, fluoranthene, chryzene, benz[a]anthracene, benz[a]pirene, benz[a]fluoranthene, benz[ghi]perylene), PCBs (congeners: 28, 52, 101, 118, 138, 153, 180) and heavy metal content (Zn, Cu, Pb, Ni, Hg, Cd, Cr) in mg/kg

^a^(Journal of Laws 2002, no. 165, pos. 1359)

^b^“Meters below ground surface”

^c^Presented uncertainty values represent expanded uncertainty at coefficient k = 2 and confidence level at 95 %

^d^Referring to the sum of aliphatic, naphthene and aromatic hydrocarbons containing 6–12 carbon atoms in the compound, including mono-aromatic compounds BTEX (benzene, toluene, ethylobenzene and xylene)

^e^Referring to the sum of aliphatic, naphthene and aromatic hydrocarbons containing 12–35 carbon atoms and above in the compound, including polycyclic aromatic hydrocarbons PAHs (naphthalene, phenanthrene, anthracene, fluoranthene, chrysene, benz(a)anthracene, benz(a)pyrene, benz(a)fluoranthene, benz(ghi)perylene)

^f^Referring to the sum of PAH concentrations, i.e. naphthalene, fenantrene, antracene, fluorantrene, chrysene, benz(a)anthracene, benz(a)pyrene, benz(a)fluoranthene, benz(ghi)perylene

^g^PCBs—referring to polychlorane diphenyls, polychlorane triphenyls, monometylotetrachlorodiphenylometane, monometylodichlorodiphenylometane, monometylofibromodiphenylometane and mixtures containing any of these substances in amounts exceeding 0.005 % total weight (Journal of Laws 2001, no. 62, pos. 627; Journal of Laws 2002, no. 165, pos. 1359)

The highest contents of oil-derived substances were noted in the soil sample from Białystok Fabryczny (sum of petrols: 134.1 ± 40.2 mg/kg; sum of mineral oils: 2520.0 ± 730.8 mg/kg). PAH (20.5 ± 4.9 mg/kg) and PCB (0.116 ± 0.046 mg/kg) contents were the highest in the soil from railway tracks at Białystok Fabryczny. In the soil from Siemianówka there were medium contents of oil-derived substances (sum of petrols: 92.1 ± 27.6 mg/kg; sum of mineral oils: 1099.0 ± 318.7 mg/kg) and PAHs (3.3 ± 0.8 mg/kg). In comparison to samples from Białystok Fabryczny and Siemianówka, the soils from Waliły had lower contents of oil-derived substances (sum of petrols: 13.4 ± 4.0 mg/kg; sum of mineral oils: 831.5 ± 241.1 mg/kg) and PAHs (2.3 ± 0.6 mg/kg). In soil samples from Siemianówka and Waliły there were lower contents of PCBs (<0.021 mg/kg) than in the sample from Białystok Fabryczny (Table 4). Heavy metals were measurable in all samples. Highest concentrations often were found in the soil from Białystok Fabryczny (Zn 130 ± 10.4 mg/kg, Cu 107 ± 16.1 mg/kg, Pb 153 ± 27.5 mg/kg, and Hg 0.06 ± 0.01 mg/kg), but soil from Waliły had the highest concentrations of Ni (52 ± 12.5 mg/kg) and Cr (70 ± 14.7 mg/kg). Pesticide concentrations exceeding the lower detection limits were not observed in any of the samples.

In relation to Polish and EU legal limits for soil (Table 4), only the content of mineral oils in soils from Białystok Fabryczny after adding the estimated uncertainty (∑mineral oils 2520.0 ± 730.8 mg/kg) almost exceeded the admissible concentrations for soils from industrial and communication areas. The admissible content of ∑mineral oils is 3000 mg/kg (Journal of Laws 2002, no. 165, pos. 1359). The contents of the remaining substances in the examined soils from railway tracks did not exceed the admissible values determined for soils from industrial and communication areas.

## Discussion

### Biotests

The complex evaluation of the influence of pollution of the soils from railways tracks on the test organisms was conducted using a set of biotests—Phytotoxkit, Ostracodtoxkit, Daphtoxkit and Microtox. The application of organisms with variable sensitivity and representing different trophic levels has allowed determining the two most toxic soils from railway tracks to be those from Siemianówka and Białystok Fabryczny (Table 5). The explicit reaction of all applied test organisms points to the significantly high toxicity of soils from railway tracks from Białystok Fabryczny and Siemianówka. The obtained result may indicate the presence of synergistic actions of pollutants from soils on the test organisms. It seems that a single action of the particular pollutants would not have resulted in such effect. In turn, the joint action of several pollutants apparently resulted in a toxic effect on the organisms. This hypothesis requires further study.

**Table 5:** Results of biotests for the analysed soils from railway tracks from stations in north-eastern Poland

Station	Phytotoxkit			Ostracodtoxkit	Daphtoxkit	Microtox
	*L. sativum*	*S. alba*	*S. saccharatum*	*H. incongruens*	*D. magna*	*V.* *fischeri*
Białystok Fabryczny	**+**	**+**	**+**	**+**	**+**	**+**
Siemianówka	**+**	**+**	**+**	**+**	**+**	**+**
Hajnówka	**+**	−	−	−	−	−
Iława Główna, siding	**+/**−	−	**+/**−	−	**+/**−	−
Iława Główna, bottle-washer	**+/**−	−	−	**+/**−	−	**+/**−
Waliły	**+/**−	−	**+/**−	−	−	−

The symbols refer to soils with high toxicity (+), soils with medium toxicity (**+/−**), and non-toxic soils (−) with regard to the test organisms applied in particular biotests

Soils from the remaining stations, regardless the applied biotest, showed low influence on the test organisms or did not have any influence at all in comparison to the control batch (Table 5).

Among the examined media, soil from the railway tracks at the Waliły station had the lowest influence on the test organisms (Table 5). Thus it can be assumed that this medium is not toxic to organisms.

The basic soil properties of the railway subsoils from this region were described in Galera et al. (2011), which was a part of our previous research. Many railway subsoils in north-eastern Poland were described in Galera et al. (2011), including those from Białystok Fabryczny and Siemianówka. Crushed stone is the material used for the bedding layer in the railway embankment. Types of rocks often used as a crushed stone include granite, porphyry, basalt, gneiss or marble. In Poland, the most commonly used crushed stone is porphyry. Trackbed are also sprinkled with a layer of river sand for instance. After years of use of the railway track, the pH for most toxic railway subsoils from Białystok Fabryczny and Siemianówka amounted to 7.80 and 7.78 in H_2_O, and from 7.80 to 7.57 in KCl, respectively. The N content in substrate from Białystok Fabryczny was 0.341 % and from Siemianówka 0.215 %. The available P content in railway subsoils from Białystok Fabryczny and Siemianówka amounted to 36.391 mg 100 g^−1^ (very high content) and 6.493 mg 100 g^−1^ of soil, respectively. The exchangeable cation levels were relatively similar in all the investigated areas, decreasing in the order Ca^2+^ > Mg^2+^ > K^+^ > Na^+^. For railway subsoils from Białystok Fabryczny and Siemianówka exchangeable cation levels amounted to: Ca—400.441 and 214.542 mg 100 g^−1^, Mg—14.546 and 6.313 mg 100 g^−1^, K—11.592 and 1.885 mg 100 g^−1^, Na—2.176 and 0.896 mg 100 g^−1^, respectively (Galera et al. 2011). The chemical features of the subsoil from Białystok Fabryczny and Siemianówka do not seem to be a factor influencing to a high toxicity of this two soils for test organisms which was used in biotests (Table 6).

**Table 6:** Chemical parameters of the subsoil from some railway areas in north-eastern Poland (Galera et al. 2011)

Railway areas	pH (H_2_O)	pH (KCl)	Total, N (%)	Available P, mg 100 g^−1^	Mg, mg 100 g^−1^	Ca, mg 100 g^−1^	Na, mg 100 g^−1^	K, mg 100 g^−1^
Białowieża Towarowa	7.69	7.56	0.279	1.030	12.863	363.585	1.507	6.950
Narewka—railway siding	8.00	7.94	0.084	1.399	6.410	254.767	0.318	1.869
Nowosady—forest	7.77	7.50	0.374	4.894	7.371	213.033	0.405	1.775
Straszewo—forest loading ramp	7.88	7.82	0.090	2.432	4.596	190.917	0.428	2.081
Białowieża Pałac	7.72	7.53	0.134	1.723	16.567	346.225	1.768	6.630
Nowosady—grassland	7.73	7.53	0.360	4.340	29.380	261.450	0.573	0.466
Lewki	7.98	7.76	0.098	5.082	9.363	304.100	1.694	7.446
Kołaki—small station in the forest	7.58	7.24	0.317	4.467	9.363	340.775	1.887	11.696
Hajnówka	7.37	7.23	0.368	4.070	10.642	331.350	2.562	7.304
Siemianówka	7.78	7.57	0.215	6.493	6.313	214.542	0.896	1.885
Sokoły—town station	7.98	7.68	0.242	10.010	7.550	379.983	2.322	10.067
Białystok Fabryczny	7.80	7.39	0.341	36.391	14.546	400.441	2.176	11.592

All biotests applied in this research have been used earlier in the analysis of soils containing such pollutants as oil-derived products, PAHs, PCBs, herbicides or heavy metals (Aelion and Davis 2007; Czerniawska-Kusza et al. 2006; Davoren et al. 2005; Hund-Rinke et al. 2002; Plaza et al. 2005, Plaza et al. 2010; Põllumaa et al. 2004; Sekutowski and Sadowski 2009), i.e. substances considered as typical pollutants linked with railway transport (Wiłkomirski et al. 2011, 2012). Thus, they are relevant tools in the assessment of the soil condition on railway tracks.

### Pollutants

A mixture of different pollutants was detected in the examined soils from railway tracks, including oil-derived products, PAHs, PCBs and heavy metals. It is, however, worth noting that despite the determined toxicity of soils from railway tracks, the detected pollution levels do not exceed admissible concentrations in soils, ascertained in Polish Law for industrial and communication areas (Journal of Laws 2002, no. 165, pos. 1359). These limits are consistent with EU regulations (Table 4).

Results of chemical analyses confirm a synergistic effect of low doses (in the range of the existing limits) of several different pollutants, which as a result caused the toxic influence of soils from the railway tracks at Białystok Fabryczny and Siemianówka on the test organisms.

### Oil-derived substances

Among the examined soils, the highest content of oil-derived substances was determined in the most toxic soil from the railway tracks at Białystok Fabryczny, which is, however, within admissible levels for communication areas. A significantly high content of these substances was also noted in the soil from Siemianówka (also in the range of the existing limits). It seems that the main cause of such high toxicity of soils from these two stations with regard to the test organisms in all biotests were high contents of oil-derived substances, although not exceeding the admissible values. The content of mineral oils from soil at Białystok Fabryczny was at the boundary of admissible values determined for soils from industrial and communication areas (Journal of Laws 2002, no. 165, pos. 1359). In comparison to samples from Białystok Fabryczny and Siemianówka, the non-toxic soil from Waliły contained much lower contents of oil-derived products (Table 4).

The presence of oil-derived substances in railway areas (Burkhardt et al. 2008; Malawska and Wiłkomirski 1999, 2001a; Wiłkomirski et al. 2011, 2012) may pose environmental hazards. Substances transported in the soil cause changes of its physical–chemical and biological properties. These changes cause effects such as necrosis of animal organisms dwelling in the topsoil and their growth anomalies, conducting of water and mineral soils, as well as morphological changes (Besalatpour et al. 2008; Gierak 1995; Wyszkowska and Kucharski 2000; Ziółkowska and Wyszkowski 2010).

The contents of oil-derived substances in the most toxic soils can be compared with the results obtained by Okop and Okorie (2013), who studied soils after a huge oil spill in the Niger River delta. Although the level of the oil-derived products was much lower there in comparison to those in the examined soils from Białystok Fabryczny and Siemianówka, Okop and Okorie (2013) have recommended careful monitoring and recultivation of the polluted areas due to the possible appearance of hazardous environmental effects. Based on this example and the conducted biological and chemical analyses it can be assumed that pollution by oil-derived products of soils from railway tracks at Białystok Fabryczny and Siemianówka may cause environmental hazard despite the fact that the admissible values for communication areas have not been exceeded.

### PAHs and PCBs

Both PAHs and PCBs are common pollutants of railway areas (Burkhardt et al. 2008; Malawska and Wiłkomirski 1999, 2001a, 2001b; Moret et al. 2007; Thierfelder and Sandström 2008; Wiłkomirski et al. 2011, 2012). In this paper, the highest PAH and PCB contents among the examined media have been noted in the soil from Białystok Fabryczny, whereas medium values were observed in the soil from Siemianówka—in each case the values were within the admissible ranges. Lower PAH and PCB contents were noted in the soil from Waliły (Table 4). These results can be compared with the reports by Malawska and Wiłkomirski, who examined soils and plants from the Iława Górna station; the area was considered significantly polluted by PAHs (Malawska and Wiłkomirski 2001b; Wiłkomirski et al. 2011). In relation to samples from Iława Górna, the PAH content in soils from Białystok Fabryczny, Siemianówka and Waliły was higher, particularly in Białystok Fabryczny. Although these values do not exceed Polish legal limits for soil (Journal of Laws 2002, no. 165, pos. 1359) (Table 4), this may have been another factor influencing high toxicity of soils from Białystok Fabryczny.

Transfer of PAHs to soil and plants is likely; it decreases along with increasing distance from the pollution source, i.e. railway ties (Malawska and Wiłkomirski 2001b; Moret et al. 2007; Wiłkomirski et al. 2011, 2012). This poses potential hazard to organisms. Malawska and Wiłkomirski (1999, 2001a) presented the PCB pollution in soils and plants near railway tracks based on data from the railway junctions near Iława Górna and Tarnowskie Góry and two important railway lines Warszawa-Gdańsk (area near Iłowo) and Katowice-Gdynia (area near Warlubie and Laskowice), where higher PCB values have been noted in soils and plants growing in the area. Comparison of data from these reports with the results of the present research indicate that only the soil from Białystok Fabryczny achieved the same pollution level by PCB compounds. These values do not exceed the admissible values (Journal of Laws 2002, no. 165, pos. 1359) (Table 4). Highly chlorinated PCBs are adsorbed on the soil surface with a high content of organic substances, e.g. polluted by oil-derived substances—in this case PCBs do not infiltrate inwards (Malawska and Wiłkomirski 1999, 2001a; Wiłkomirski et al. 2012). Due to the fact that soils from Białystok Fabryczny and Siemianówka contain significant amounts of oil-derived substances, it is possible that the recognized PCBs were adsorbed on the surface of the examined soils and did not infiltrate inwards. However, they still pose potential hazard to organisms.

### Heavy metals

The main pollutants emitted to the environment by railway transport are heavy metals and their content decreases with increasing distance from the railway track. They may infiltrate from soils to plants; thus, there is a risk of their transportation to higher trophic levels. The method of binding of heavy metals in soil and thus their bioavailability depend e.g. on the metal form, substrate composition and its physical and chemical properties (Burkhardt et al. 2008; Hławiczka 2008; Kabata-Pendias and Pendias 1999; Liu et al. 2009; Malawska and Wiłkomirski 2000, 2001b; Wiłkomirski et al. 2011, 2012; Zhang et al. 2012). The presence of heavy metals was noted in all examined soils—the highest concentration for most was found in soil from Białystok Fabryczny. Admissible levels were not exceeded (Journal of Laws 2002, no. 165, pos. 1359). A medium content of heavy metals was noted in soil from Waliły and the lowest in soil from Siemianówka (Table 4).

The content of heavy metals in soils from Białystok Fabryczny, Siemianówka and Waliły can be compared with the results obtained by Liu et al. (2009) and Zhang et al. (2012), where the determined level of various heavy metals in soils from railway areas was identical or even lower than in soils from Białystok Fabryczny and Waliły (e.g. Cu, Zn and Pb). Soil from Siemianówka was characterized by lower pollution by heavy metals in comparison to the cited reports. Earlier studies of the Białystok Fabryczny and Siemianówka areas (Galera et al. 2011) indicate similar or slightly higher values of Zn and Cu in the soil in comparison to this report. However, it was assumed that such contents of heavy metals do not negatively influence the growth of plants in the area.

### Pesticides

The presence of pesticides in soils is linked with their presence in plants, water pollution, possible effects on soil organisms and soil fertility (Różański 1992; Walker et al. 2002; Alloway and Ayres 1999; Seńczuk 2002). In the studied soils, pesticide concentrations were within the admissible values despite the fact that such substances are commonly considered to occur as pollutants in railway areas (Burkhardt et al. 2008; Schweinsberg et al. 1999; Wiłkomirski et al. 2011, 2012). Roundup—a non-selective herbicide with *N*-(phosphomethyl)glycine (i.e. glyphosate) as the active substance—is often used in railway areas. After application, it is rapidly degraded by soil organisms (Material Safety Data Sheet Roundup 360 SL; Schweinsberg et al. 1999). Most other pesticides are also degraded in the soil. Fast degradation is the possible cause of the lack of the substances in the studied soils.

## Summary

The toxic effect of soils from railway tracks at Białystok Fabryczny and Siemianówka on the test organisms representing different trophic groups likely was caused by the synergistic simultaneous effect of several pollutants, whose content in all examined soils was within the admissible levels for soils from communication areas. This result clearly points to the need of conducting ecotoxicological analyses along with chemical analysis during environmental assessments. For example, the soil from Waliły with a much lower content of most pollutants in comparison to soils from Białystok Fabryczny and Siemianówka did not have any toxic effect on the test organisms.

Based on data obtained from biological and chemical analyses it can be concluded that railway transport may cause potential hazard to the natural environment to a larger degree than hitherto assumed. Monitoring of railway areas which might cause a significant effect on the environment seems a justified decision.

## Conclusions

The conducted analyses have allowed obtaining the following results:

- Soil toxicity along railway tracks is highly variable.
- High toxicity of soils from Białystok Fabryczny (the highest toxicity) and Siemianówka has been determined. The soils pose definite hazard on the test organisms from all trophic levels.
- Oil-derived substances, heavy metals, PAHs and PCBs were found in all examined soils from railway tracks. Despite the fact that the pollutants did not exceed admissible pollution levels, the biological analyses have allowed to determine the most toxic soils.
- The obtained result may indicate the presence of synergistic, harmful effect of pollutants in soils from railway tracks on test organisms from all trophic levels. This effect was probably the main cause of high toxicity of soils from railway tracks at Białystok Fabryczny and Siemianówka.
- Based on the obtained data (biological and chemical) it has been assumed that railway transportation may cause hazard to the natural environment.
